# P2X4 Receptor-Dependent Ca^2+^ Influx in Model Human Monocytes and Macrophages

**DOI:** 10.3390/ijms18112261

**Published:** 2017-10-27

**Authors:** Janice A. Layhadi, Samuel J. Fountain

**Affiliations:** School of Biological Sciences, University of East Anglia, Norwich Research Park, Norwich NR4 7TJ, UK; j.layhadi@uea.ac.uk

**Keywords:** P2X4, ATP, calcium, monocyte, macrophage, purinergic receptor

## Abstract

Monocytes and macrophages express a repertoire of cell surface P2 receptors for adenosine 5′-triphosphate (ATP) a damage-associated molecular pattern molecule (DAMP), which are capable of raising cytoplasmic calcium when activated. This is achieved either through direct permeation (ionotropic P2X receptors) or by mobilizing intracellular calcium stores (metabotropic P2Y receptors). Here, a side-by-side comparison to investigate the contribution of P2X4 receptor activation in ATP-evoked calcium responses in model human monocytes and macrophages was performed. The expression of P2X1, P2X4, P2X5 and P2X7 was confirmed by qRT-PCR and immunocytochemistry in both model monocyte and macrophage. ATP evoked a concentration-dependent increase in intracellular calcium in both THP-1 monocyte and macrophages. The sarco/endoplasmic reticulum Ca^2+^-ATPase inhibitor thasigargin (Tg) responses to the maximal ATP concentration (100 μM) in THP-1 monocytes, and responses in macrophage were significantly attenuated. Tg-resistant ATP-evoked calcium responses in the model macrophage were dependent on extracellular calcium, suggesting a requirement for calcium influx. Ivermectin (IVM) potentiated the magnitude of Tg-resistant component and slowed the decay of response in the model macrophage. The Tg-resistant component was attenuated by P2X4 antagonists 5-BDBD and PSB-12062 but not by the P2X1 antagonist Ro0437626 or the P2X7 antagonist A438079. shRNA-mediated P2X4 knockdown resulted in a significant reduction in Tg-resistant ATP-evoked calcium response as well as reduced sensitivities towards P2X4-specific pharmacological tools, IVM and PSB-12062. Inhibition of endocytosis with dynasore significantly reduced the magnitude of Tg-resistant component but substantially slowed decay response. Inhibition of calcium-dependent exocytosis with vacuolin-1 had no effect on the Tg-resistant component. These pharmacological data suggest that P2X4 receptor activation contributed significantly towards the ionotropic calcium response evoked by ATP of the model human macrophage.

## 1. Introduction

The release of adenosine 5′-triphosphate (ATP) as a signaling molecule by activated leukocytes, platelets and apoptotic cells is crucial for the induction of a variety of physiological responses across different cell types [1,2]. ATP is able to mediate these biological processes through the activation of purinergic P2 receptors: P2X (ligand-gated ion channels) or P2Y (G-protein coupled receptors) [3,4]. To date, seven mammalian P2X receptor subunits (P2X1–P2X7) have been identified with widespread distribution throughout neuronal and non-neuronal tissues [5]. The expression of P2X receptors has been reported in immune cells such as monocytes and macrophages, with P2X4 and P2X7 being the pre-dominant subunits expressed [6]. Although significant research has been performed in the past decade to elucidate a role of P2X7 receptor in the immune system, the same cannot be said for the P2X4 receptor. Research on the P2X4 receptor has been greatly hampered by the lack of selective P2X4 receptor antagonists as well as the possible complicating presence of the P2X7 receptor [7]. In macrophages and microglia, P2X4 receptors have been shown to co-express with P2X7 receptors, although they do not appear to share the same downstream signaling pathways [8,9].

Although the role of P2X4 receptors in monocytes and macrophages is not clearly defined within the literature, P2X4 expression has been shown to be upregulated following nerve damage in microglia [10] and, in macrophages, P2X4 has been reported to mediate prostaglandin E2 (PGE2) release [11]. Studies by Li and Fountain (2012) in THP-1 monocytes also illustrated that P2X4 contributes towards the ATP-evoked Ca^2+^ response and that its activity is suppressed by cholesterol lowering drug, fluvastatin [12].

In the present study, we have performed a side-by-side comparison of the expression of P2X receptors in model human THP-1 monocytes and macrophages through the use of qRT-PCR and immunocytochemistry. Though evidence of functional P2X4 can be revealed by whole-cell patch-clamps, studies evaluating the role of P2X receptors in intact cells are limited. To this end, we investigated the contribution of P2X4 receptor activation towards ATP-evoked intracellular Ca^2+^ response, and this was assessed in the two cell models through intracellular Ca^2+^ measurements. Our findings allowed the identification of P2X4 contribution of towards ATP-evoked Ca^2+^ influx in model human monocytes and macrophages, while also assessing the reliability of commercially available pharmacological tools for the study of P2X4.

## 2. Results

**Figure 1:**
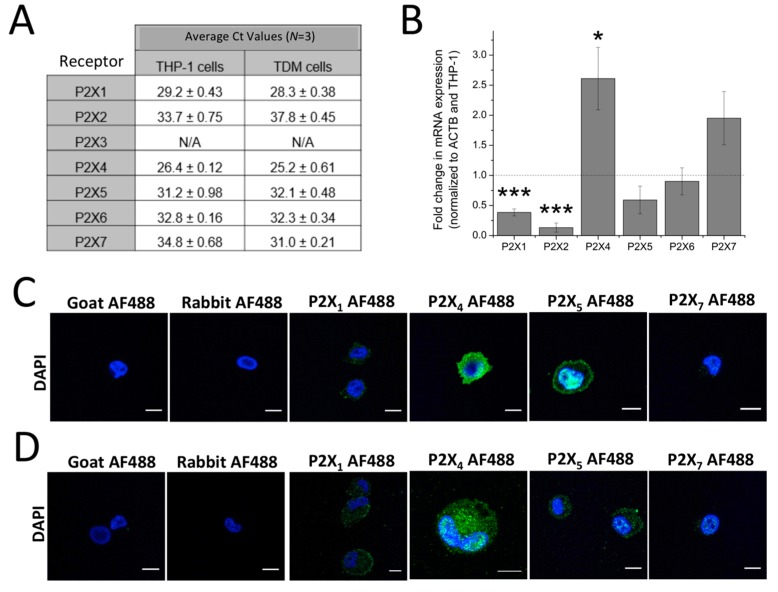
Expression of P2X receptors in THP-1 monocytes and THP-1-differentiated macrophages. (**A**) table showing qRT-PCR Ct values to identify which P2X genes are expressed in THP-1 cells and THP-1 differentiated macrophages. Ct values above 35.0 were considered absent (*n* = 3). Undetected genes are represented as N/A; (**B**) fold change in mRNA expression of P2X receptor genes in THP-1 differentiated macrophages, normalized to β-actin and THP-cells (*n* = 3). Asterisks include significant changes towards control (*** *p* < 0.001, * *p* < 0.05); (**C**,**D**) immunocytochemistry of P2X receptor expression in THP-1 monocytes and THP-1 differentiated macrophages, respectively, as observed under confocal microscopy. Blue represents 2-4(amidinophenyl)-1*H*-indole-6-carboxamidine (DAPI) nuclear stain while green represents human P2X receptor conjugated with AF488. Scale bar represents 10 μm. Images are representative of *n* = 3 replicates. TDM: THP-1 differentiated macrophages.

P2X1, P2X4, P2X5 and P2X7 are expressed at the mRNA and protein level in THP-1 monocytes and macrophages. Expression of P2X receptors in human THP-1 monocytes and THP-1 differentiated macrophages were investigated at the mRNA and protein level. qRT-PCR analysis identified mRNA expression of all P2X genes with the exception of P2X2 in THP-1 and P2X2 and P2X3 in THP-1 differentiated macrophages (Figure 1A). Fold change in mRNA expression was also quantified and showed that, across three replicates, P2X1 and P2X2 mRNA expression was found to be downregulated (P2X1: 0.38 ± 0.058 fold; *n* = 5; *p* < 0.001 and P2X2: 0.13 ± 0.074 fold; *n* = 5; *p* < 0.001), while P2X4 were found to be upregulated (2.61 ± 0.52 fold; *n* = 5; *p* < 0.05) in THP-1-differentiated macrophages compared to THP-1 monocytes (Figure 1B). Using immunocytochemistry, protein expression of P2X receptors was also studied. P2X1, P2X4, P2X5 and P2X7 were all expressed in both THP-1 monocytes and THP-1-differentiated macrophages (Figure 1C,D, respectively).

### 2.1. ATP-Evoked Intracellular Ca^2+^ Responses in THP-1 Monocytes and Macrophage Models

**Figure 2:**
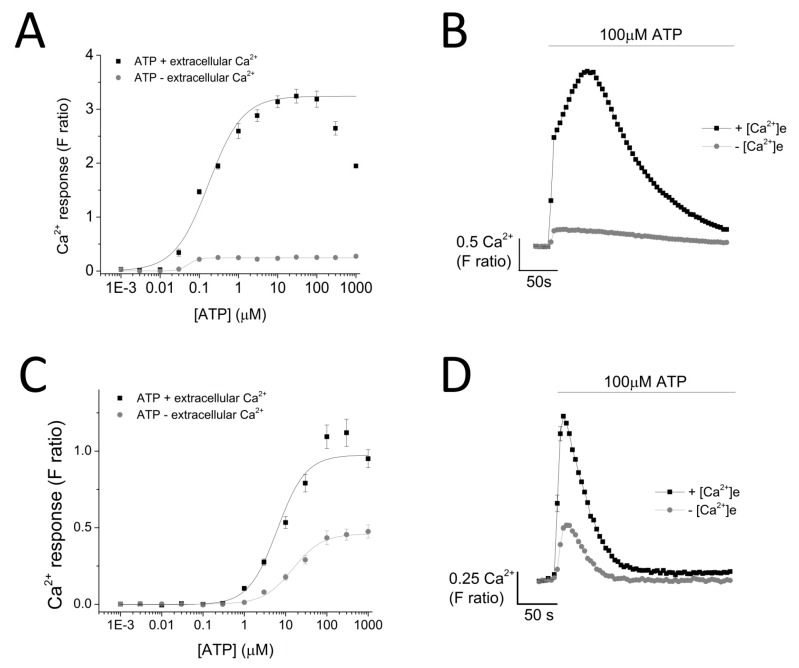

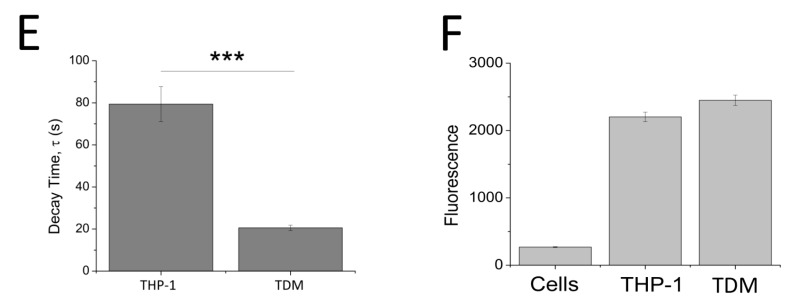
ATP-evoked intracellular Ca^2+^ responses in THP-1 monocytes and THP-1 differentiated macrophages. Concentration response curve of ATP (0.01–1000 μM) in the presence and absence of extracellular Ca^2+^ and its representative traces at 100 μM ATP in: (**A**,**B**) THP-1 cells (*n* = 3) and (**C**,**D**) THP-1 differentiated macrophages (*n* = 3); (**C**) bar chart representing decay kinetics of THP-1 cells (*n* = 12) and THP-1 differentiated macrophages (*n* = 6); (**D**,**E**) difference in decay time of 100 μM-evoked Ca^2+^ response in THP-1 versus TDM cells. Tau (τ) is the time constant calculated from fitting a single exponential decay to the falling phase of Ca^2+^ response (*n* = 5); (**F**) cell quantification assay using nuclear stain H-33342. Asterisks include significant changes towards control (*** *p* < 0.001, *n* = 4). TDM: THP-1 differentiated macrophages.

ATP evoked a concentration-dependent increase in intracellular Ca^2+^ response in both THP-1 monocytes (EC50 = 0.174 ± 0.01 μM with extracellular Ca^2+^ and EC50 = 0.051 ± 0.005 μM without extracellular Ca^2+^) (Figure 2A,B) and THP-1-differentiated macrophages (EC50 = 6.25 ± 1.92 μM with extracellular Ca^2+^ and EC50 = 14.9 ± 0.88 μM without extracellular Ca^2+^; *n* = 6) (Figure 2C,D). Response to ATP (100 μM) was biphasic; in both cell types, the response involved an initial rapid increase in intracellular Ca^2+^ response, which peaked and was followed by a decay phase. While the response decayed back to the baseline level in THP-1-differentiated macrophages, the decay response remained at an elevated phase in THP-1 monocytes (Figure 2B,D). Two striking differences were observed in the phenotypic features of the ATP-evoked Ca^2+^ response in the two cell models: (1) peak magnitude was significantly higher in THP-1 monocytes compared to THP-1-differentiated macrophages (F ratio 3.24 ± 0.127 THP-1 monocytes vs. 1.09 ± 0.077 THP-1-differentiated macrophages; *n* = 12 and 8; *p* < 0.001; Figure 2B) and (2) decay response. The decay time was quantified for both cells and illustrated a much faster decaying response in THP-1-differentiated macrophages (84.0 ± 5.28 s THP-1 monocytes vs. 18.64 ± 2.18 s THP-1-differentiated macrophages; Figure 2E). Cell quantification throughout the intracellular Ca^2+^ assay using Hoechst stain H33342 showed no significant differences in the number of cells used throughout the experiments, eliminating the possibility that the difference in peak magnitude in the two cell types were due to difference in cell number (Figure 2F).

### 2.2. Dependency of ATP-Evoked Ca^2+^ Response on ER Calcium Store

**Figure 3:**
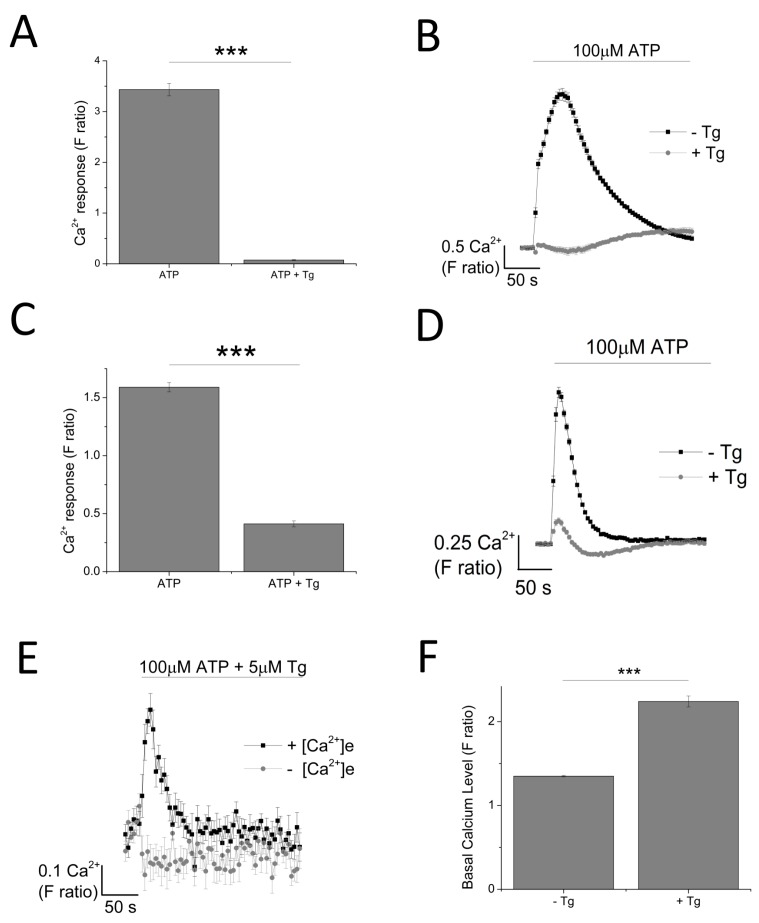
Dependency of ATP-evoked Ca^2+^ response on the endoplasmic reticulum Ca^2+^ store. The effect of 100 μM ATP in the presence and absence of 5 μM Tg in: (**A**,**B**) THP-1 monocytes (*n* = 3) and (**C**,**D**) THP-1 differentiated macrophages (*n* = 6); (**E**) time-response curve of the effect of Tg on 100 μM ATP in the presence and absence of extracellular Ca^2+^ (*n* = 6); (**F**) effect of 5 μM Tg on basal Ca^2+^ level in THP-1 differentiated macrophages (*n* = 6). Asterisks include significant changes towards control (*** *p* < 0.001).

As ATP evoked a global intracellular Ca^2+^ response, mediated by both the activation of P2X and P2Y receptors, a sarco/endoplasmic reticulum Ca^2+^-ATPase inhibitor, Thapsigargin (Tg), was employed to eliminate metabotropic responses. Tg depletes intracellular stores of Ca^2+^ resulting in a raise in cytosolic Ca^2+^ level (F ratio 1.35 ± 0.0082 without Tg vs. 2.24 ± 0.065 with Tg; *n* = 6; *p* < 0.001; Figure 3). Pre-treatment of cells with 5 μM Tg completely attenuated ATP-evoked intracellular Ca^2+^ response in THP-1 monocytes (Figure 3A,B) while significantly inhibiting ATP-evoked intracellular Ca^2+^ response in THP-1-differentiated macrophages (88.7 ± 3.29% inhibition; *n* = 6; *p* < 0.001) (Figure 3C,D). Despite a significant inhibition with Tg, the results showed that 11.3 ± 3.29% (*n* = 6) of the ATP-evoked Ca^2+^ response appeared to be resistant to Tg. In the absence of extracellular Ca^2+^, this Tg-resistant Ca^2+^ response was completely abolished, implying that it is dependent on Ca^2+^ influx (Figure 3E).

### 2.3. P2X4, but not P2X1 or P2X7, Receptor Activation Mediates the Ionotropic Calcium Response in Model Human Macrophage

**Figure 4:**
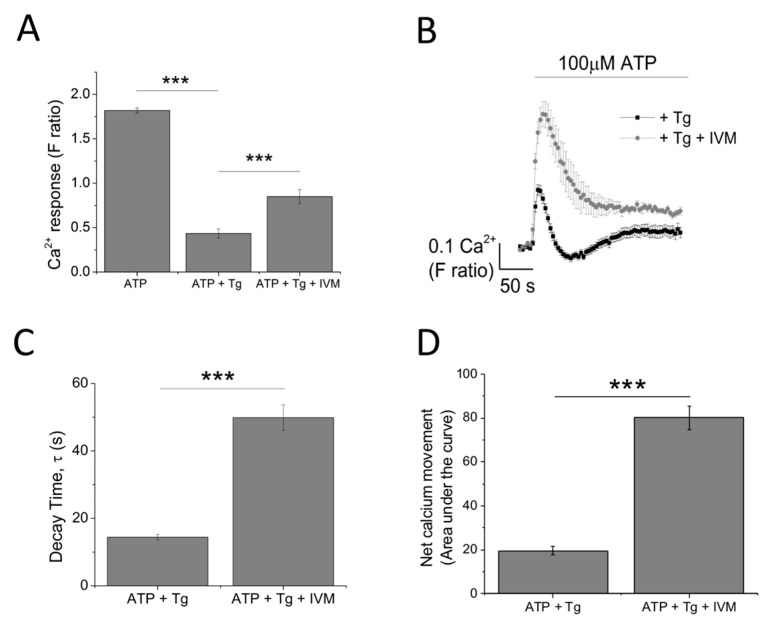
Investigating the contribution of P2X_4_ receptor using the positive allosteric modulator ivermectin. (**A**,**B**) effect of 3 μM IVM on Tg-resistant Ca^2+^ response represented as bar chart and time-response curve, respectively (*n* = 6). The effect of 3 μM ivermectin (IVM) on: (**C**) decay kinetics (*n* = 6) and (**D**) net calcium movement as quantified by area under the curve (*n* = 6) of THP-1 differentiated macrophages. Asterisks include significant changes towards control (*** *p* < 0.001).

**Figure 5:**
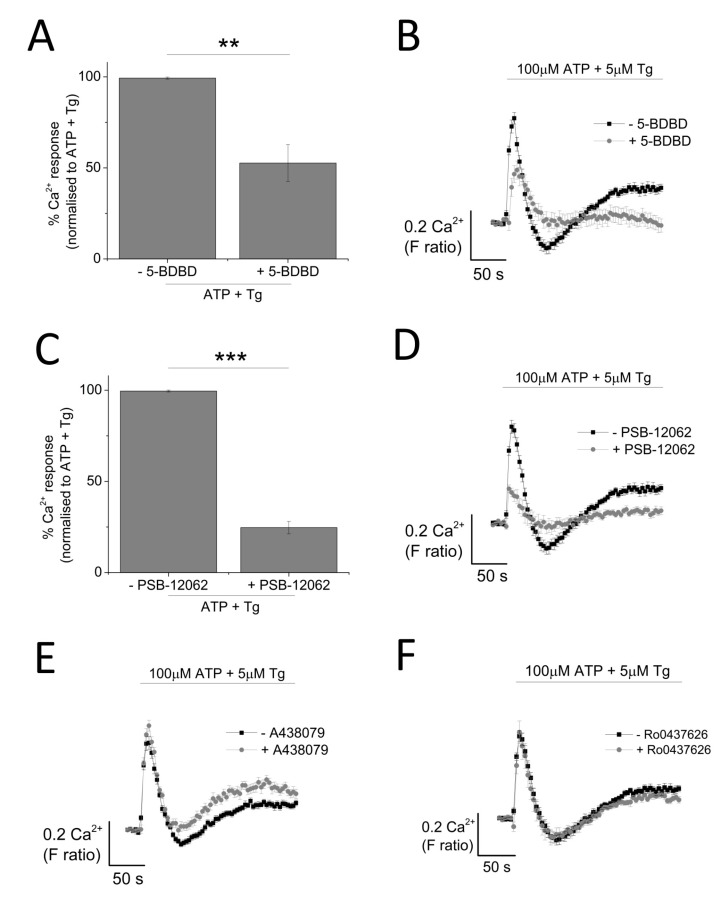
Effect of selective P2X receptor antagonists on Tg-resistant ATP-evoked Ca^2+^ response in THP-1 differentiated macrophages. (**A**,**B**) effect of P2X_4_ receptor antagonist (10 μM 5-BDBD) on magnitude of Ca^2+^ response and net calcium movement on Tg-resistant Ca^2+^ response (*n* = 3); (**C**,**D**) effect of P2X_4_ receptor antagonist (10 μM PSB-12062) on magnitude of Ca^2+^ response and net calcium movement on Tg-resistant Ca^2+^ response (*n* = 3); (**E**) effect of P2X_7_ receptor antagonist (5 μM A438079) represented as time response traces (*n* = 3); (**F**) effect of P2X_1_ receptor antagonist (30 μM Ro0437626) represented as time response traces (*n* = 3). Asterisks include significant changes towards control (*** *p* < 0.001, ** *p* < 0.01).

The contribution of P2X4 receptor activation towards Tg-resistant ATP-evoked Ca^2+^ response was studied using a combination of pharmacological tools. Pre-treatment of cells with P2X4-specific positive allosteric modulator (ivermectin (IVM); 3 μM) resulted in the potentiation of peak magnitude of Tg-resistant Ca^2+^ response by 2.1 fold (F ratio 0.42 ± 0.025 without IVM to 0.90 ± 0.042 with IVM; *n* = 6; *p* < 0.001) (Figure 4A,B). In addition to this, IVM also significantly delayed the decay kinetics by 3.67 ± 0.37 fold (14.4 ± 0.78 s without IVM to 49.9 ± 3.8 s with IVM; *n* = 6; *p* < 0.001) (Figure 4C). Selective P2X4 receptor antagonists, 5-BDBD and PSB-12062, were also used to investigate the contribution of P2X4. In the presence of Tg, both 5-BDBD and PSB-12062 (both at 10 μM) caused a significant inhibition towards the ATP-evoked Ca^2+^ response by 52.9 ± 1.99% (*n* = 3; *p* < 0.01; Figure 5A) and 78.5 ± 3.23% (*n* = 3; *p* < 0.001; Figure 5C), respectively. In addition to inhibiting the peak magnitude of the Tg-resistant Ca^2+^ response, both antagonists also abolished the second slower response observed in the presence of Tg (Figure 5B,D). Finally, pre-treatment of THP-1-differentiated macrophages with P2X1 (Ro0437626; 30 μM) or P2X7 (A438079; 5 μM) antagonists had no inhibitory effect on Tg-resistant Ca^2+^ responses (Figure 5E,F, respectively).

### 2.4. Silencing of P2X4 Abolished Tg-Resistant Calcium Response and Reduced Sensitivities Towards IVM and PSB-12062

**Figure 6:**
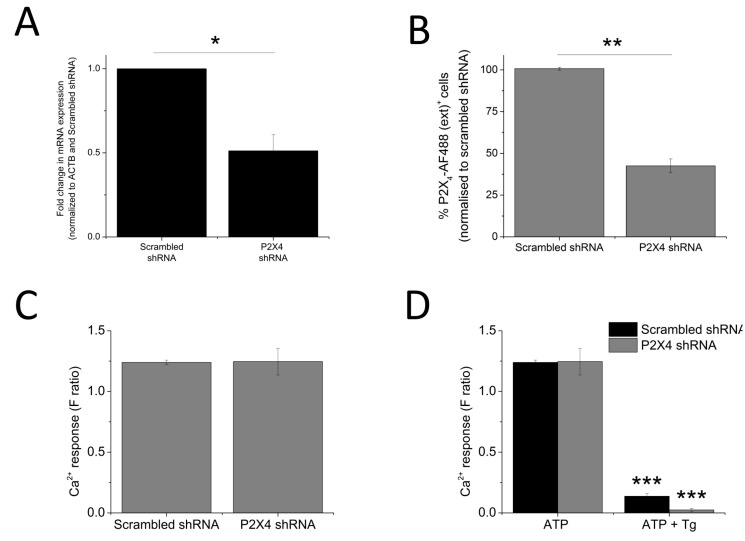
Loss of Tg-resistant ATP-evoked Ca^2+^ response in P2X_4_ knockdown THP-1 differentiated macrophages. (**A**,**B**) quantification of P2X_4_ knockdown level in THP-1 differentiated macrophages as measured using qRT-PCR for mRNA expression (*n* = 3) and flow cytometry for extracellular P2X_4_ protein expression (*n* = 3), respectively. Knockdown level was quantified against negative control scrambled cells; (**C**) ATP-evoked Ca^2+^ response at 100 μM on scrambled shRNA and P2X_4_ shRNA THP-1 differentiated macrophages (*n* = 4); (**D**) effect of Tg on 100 μM ATP response in scrambled versus P2X_4_ shRNA THP-1 differentiated macrophages (*n* = 3). Asterisks include significant changes towards control (* *p* < 0.05, ** *p* < 0.01, *** *p* < 0.001).

**Figure 7:**
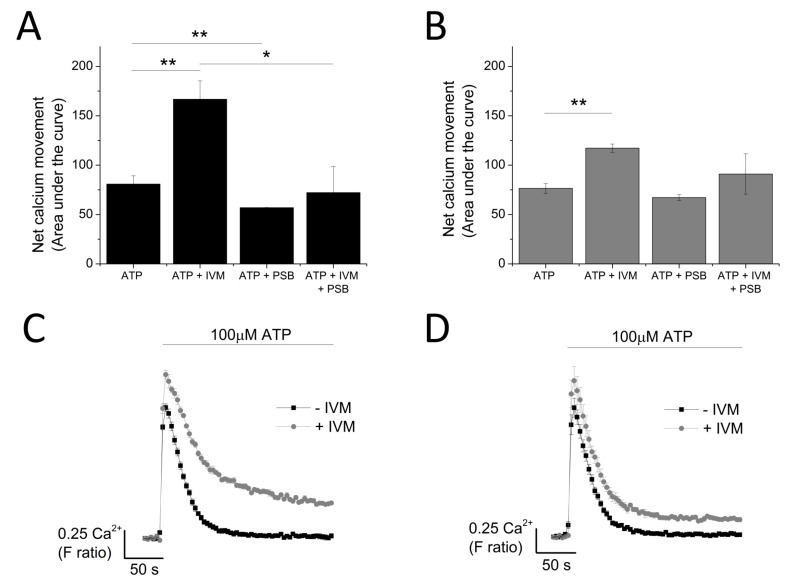
Knocking down P2X_4_ reduced the sensitivity of THP-1 differentiated macrophages to IVM and PSB-12062. The effect of 10 μM PSB-12062 on ATP response in the presence and absence of IVM in: (**A**) scrambled shRNA (*n* = 4) vs. (**B**) P2X_4_ shRNA (*n* = 4). Representative time-response curves of the effect of IVM on ATP response in: (**C**) scrambled shRNA (*n* = 4) vs. (**D**) P2X_4_ shRNA (*n* = 4). Asterisks include significant changes towards control (*** *p* < 0.001, ** *p* < 0.01, * *p* < 0.05).

The data so far illustrated that P2X4 receptors contribute towards the *T*g-resistant ATP-evoked intracellular Ca^2+^ level. To provide further confirmation, a molecular-based approach to silence the P2X4 gene was employed. The success of the gene silencing technique was confirmed by the qRT-PCR approach (48.8 ± 9.64% knockdown; *n* = 3; *p* < 0.01; Figure 6A) and flow cytometry (57.7 ± 4.37% reduction in cells expressing extracellular P2X4; *n* = 3; *p* < 0.01; Figure 6B). Despite a reduction in cell surface expression of P2X4 receptor, no significant changes in 100 μM ATP-evoked intracellular Ca^2+^ responses were observed in the P2X4-shRNA cells when compared to scrambled cells (Figure 6C). However, silencing of P2X4 significantly reduced the Tg-resistant ATP-evoked Ca^2+^ response in THP-1-differentiated macrophages (F ratio 0.14 ± 0.02 ATP + Tg scrambled shRNA vs. F ratio 0.02 ± 0.014 ATP + Tg P2X4 shRNA; *n* = 3; *p* < 0.001; Figure 6D). The effect of IVM and PSB-12062 was assessed on the P2X4 shRNA cells. When compared to scrambled negative control cells (Figure 7A,B), P2X4 shRNA cells were found to be less sensitive towards both IVM and PSB-12062, as quantified using area under the curve (Figure 7B). While IVM caused a potentiation towards the net calcium movement of scrambled shRNA cells by 2.06 ± 0.23 fold (*n* = 3; *p* < 0.01; Figure 7A,B), IVM was only able to potentiate the net calcium movement of P2X4 shRNA cells by 1.53 ± 0.055 fold (*n* = 3; *p* < 0.01; Figure 7C,D). These data provide further evidence that P2X4 receptor contributes towards Ca^2+^ influx in THP-1-differentiated macrophages and that both IVM and PSB-12062 are useful tools for the study of P2X4.

### 2.5. Targeting Receptor Trafficking to Investigate P2X4 Contribution towards Tg-Resistant Calcium Response

**Figure 8:**
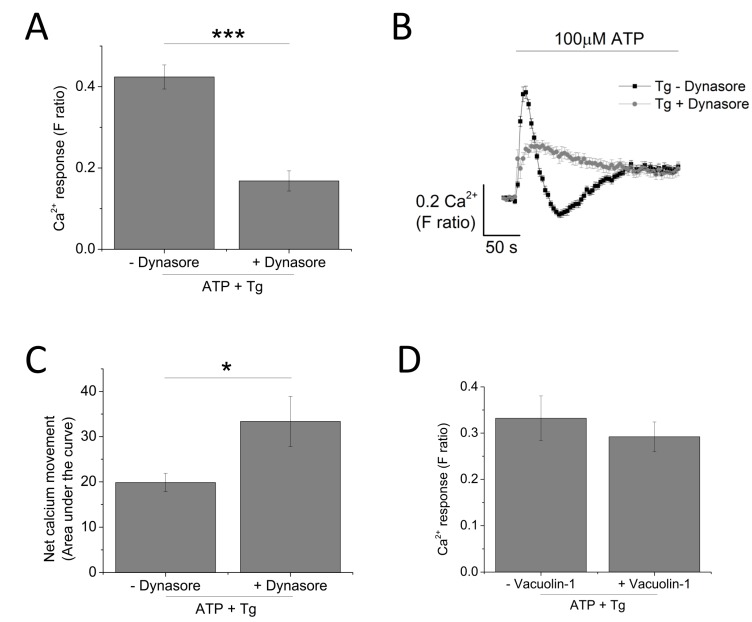
Targeting receptor trafficking to investigate contribution of P2X_4_ receptor towards ATP-evoked Ca^2+^ response in THP-1 differentiated macrophages. (**A**–**C**) effect of blocking endocytosis (80 μM dynasore) on magnitude and net calcium movement (*n* = 3) of Tg-resistant ATP-evoked Ca^2+^ response; (**D**) effect of blocking lysosomal exocytosis (1 μM vacuolin-1) on magnitude of Tg-resistant ATP-evoked Ca^2+^ response (*n* = 3). Asterisks include significant changes towards control (*** *p* < 0.001, * *p* < 0.05).

Since P2X4 receptors have been previously described to be predominantly localized within lysosomal compartments [13], targeting trafficking mechanism (dynasore to block endocytosis and vacuolin-1 to block exocytosis) would provide further evidence towards the contribution of P2X4 towards ATP-evoked Ca^2+^ response. Pre-treatment of THP-1-differentiated macrophages with dynasore (80 μM) resulted in a significant reduction of the magnitude of Tg-resistant ATP-evoked Ca^2+^ response (0.42 ± 0.17 vs. 0.17 ± 0.25; *n* = 3; *p* < 0.001; Figure 8A). However, dynasore caused a significant delay in the decay kinetics (16.2 ± 1.20 s vs. 81.5 ± 7.03 s; *n* = 3; *p* < 0.001; Figure 8B) and a significant increase in the net calcium movement as quantified by area under the curve (AUC 19.9 ± 2.02 without dynasore vs. 33.36 ± 5.56 with dynasore; *n* = 3; *p* < 0.05; Figure 8C). Pre-treatment of cells with 1 μM vacuolin-1 had no significant effect on the magnitude of the Tg-resistant ATP-evoked Ca^2+^ response in THP-1-differentiated macrophages (Figure 8D). 

## 3. Discussion

The expression of P2X receptors is widespread within the immune system, in particular P2X4 and P2X7 [6]. Despite this observation, there has been no clear evidence of the contribution of P2X4 receptor activation towards ATP-evoked Ca^2+^ influx in monocytes and macrophages. Since THP-1 monocytes and THP-1 differentiated macrophages are frequently used as an experimentally amenable model of human monocytes and macrophages [14,15,16], we utilized these model systems to investigate the aforementioned issue. Through qRT-PCR analysis, the present study illustrated that both THP-1 and THP-1 differentiated macrophages expresse all P2X receptors at the mRNA level with the exception of P2X2 for THP-1 and P2X2 and P2X3 for THP-1 differentiated macrophages. It was also interesting to observe that PMA differentiation of THP-1 into the model macrophage caused a downregulation of P2X1 and P2X2 mRNA expression while an upregulation of P2X4 mRNA expression was observed. To date, there is no reported comparison of mRNA expression level between THP-1 monocytes and THP-1 differentiated macrophages. However, our findings corroborated previously reported evidence by Myrtek et al. (2008) illustrating the expression of all P2X receptors except for P2X2, P2X3 and P2X6 [17]. Protein expression of P2X1, P2X4, P2X5 and P2X7 was further confirmed by immunocytochemistry analysis for both cell models. 

To investigate the contribution of P2X4 receptor activation towards ATP-evoked intracellular Ca^2+^ responses in THP-1 monocytes and THP-1 differentiated macrophages, we employed intracellular Ca^2+^ measurements with the aid of various pharmacological tools. ATP elicited a concentration-dependent increase in intracellular Ca^2+^ level in both cells but with significantly different response characteristics. First, the peak magnitude of ATP-evoked Ca^2+^ responses was significantly larger in THP-1 monocytes, compared to THP-1 differentiated macrophages. Secondly, decay kinetic response in THP-1 cells was found to be significantly slower than that observed in THP-1 differentiated macrophages. Although the factors underlying these differences were not investigated further, several speculations can be made. The difference in peak magnitude may be a result of the different expression of P2Y receptors expressed on the cell surface of the two cell types. This is a plausible explanation as P2Y receptor activation has previously been reported as being responsible for mediating the first initial intracellular Ca^2+^ peak response [18,19]. A second plausible explanation would be the difference in ectoenzyme CD39 expression, responsible for the breakdown of ATP into ADP and AMP. Although monocytes and macrophages are reported to express ectoenzymes CD39 and CD73, there has been no direct comparison on the level of their expression [20,21]. On the other hand, the difference in decay kinetics of the two cells in response to ATP stimulation may be a result of the different buffering capacity of Ca^2+^ of the two cells. Previous studies revealed that antibody/FcR generated phagocytosis in macrophages is a process that relies on increase in cytosolic Ca^2+^ concentration [22,23]. Therefore, as macrophages are professional phagocytes, they may be more equipped to adapt to the changes in Ca^2+^ level. 

As ATP elicits global P2 responses constituting of P2X and P2Y receptors, we utilized a SERCA inhibitor, Thapsigargin (Tg), to deplete the intracellular stores of Ca^2+^ to eliminate mobilization of Ca^2+^ from intracellular stores by P2Y receptors [24,25]. Interestingly, pre-treatment of THP-1 monocytes with 5 μM Tg completely abolished the ATP-evoked Ca^2+^ response, suggesting that it is entirely dependent on metabotropic P2Y receptor activation. The same treatment in THP-1 differentiated macrophages resulted in a significant inhibition of the ATP-evoked Ca^2+^ response, leaving a small Tg-resistant component, which was dependent on Ca^2+^ influx. Due to the lack of Tg-resistant Ca^2+^ response, we did not pursue investigation into THP-1 monocytes and focused on THP-1 differentiated macrophages instead. Our study provides evidence regarding the contribution of P2X4 receptor towards ATP-evoked Ca^2+^ response in THP-1 differentiated macrophages. This was evident from the effect observed by the use of positive allosteric modulator ivermectin (IVM) [26] and selective P2X4 receptor antagonists 5-BDBD and PSB-12062 [27,28,29,30]. Pre-treatment of THP-1 differentiated macrophages with 3 μM IVM caused a significant potentiation towards the peak magnitude of Tg-resistant Ca^2+^ response, accompanied by a significant delay in decay kinetics. It has been previously reported that the main characteristics of the IVM effect on P2X4 receptors include potentiation in magnitude and delay in decay current, although studies by Norenberg et al. (2012) illustrated that only the effect on decay kinetics is specific to P2X4 receptors [26,28]. The effects of 5-BDBD and PSB-12062 on Tg-resistant Ca^2+^ response was also consistent, in which they both significantly reduced the peak magnitude as well as abolished the second slower response. Despite similar effect in inhibition, PSB-12062 appeared to be more potent in causing an inhibition towards the Tg-resistant Ca^2+^ response, and this may be due to the nature of the antagonists. While PSB-12062 has been described as an allosteric antagonist [28], 5-BDBD is thought to act competitively, which implies that its effect may be masked due to the high agonist concentration used throughout the study (100 μM ATP). Finally, the contribution of P2X4 receptor activation towards ATP-evoked Ca^2+^ response was confirmed with a knockdown approach, which illustrated that P2X4 knockdown THP-1 differentiated macrophages lacked a Tg-resistant ATP-evoked Ca^2+^ response as well as reduced sensitivities towards IVM and PSB-12062. 

As P2X1, P2X4 and P2X7 are all expressed in the immune cells [31,32], we also investigated the contribution of P2X1 or P2X7 towards the Tg-resistant Ca^2+^ response. Selective P2X1 antagonist Ro0437626 and P2X7 antagonist A438079 had no significant inhibitory effect towards the Tg-resistant ATP-evoked Ca^2+^ response in THP-1 differentiated macrophages. The lack of contribution of P2X1 in ATP-evoked Ca^2+^ response in these cells contradict previous studies reported by Sim et al. and Wareham et al. illustrating functional evidence of P2X1 receptor in mouse macrophages [33] and human lung mast cells [31], respectively. Our qRT-PCR data illustrated that mRNA expression of P2X1 receptors in THP-1 differentiated macrophages is downregulated compared to THP-1 monocytes. It may therefore be interesting to investigate if this downregulation translates at the protein level, therefore being responsible for the lack of P2X1 contribution in these cells. Unlike P2X1, the lack of P2X7 receptor contribution towards ATP-evoked Ca^2+^ response in THP-1 differentiated macrophages is not as surprising. It is well established that 100 μM ATP used through this study is significantly below the activation threshold for P2X7 receptors (>500 μM) [34,35,36].

Altogether, the data provides evidence that P2X4 is functionally expressed in THP-1 differentiated macrophages as reflected from their contribution towards ATP-evoked Ca^2+^ response, but their functional evidence in THP-1 monocyte is lacking. Studies by Li et al. have previously reported functional P2X4 in THP-1 monocytes, although, in this case, PLC inhibitor U-73122 was used instead of Tg [12]. Furthermore, through intracellular Ca^2+^ measurements, we have showed that IVM and PSB-12062 are reliable tools for the study of P2X4, supported with findings from P2X4 knockdown cells. 

## 4. Experimental and Materials

### 4.1. Chemicals and Reagents

The following reagents were used: PSB-12062 and ATP (Sigma-Aldrich, St. Louis, MO, USA), A438079 (Abcam, Cambridge, UK), Ivermectin, 5-BDBD, Ro0437626 and Thapsigargin (all from Tocris, Bristol, UK), and Fura 2-AM (TefLabs, Austin, TX, USA).

### 4.2. Cells

THP-1 cells were obtained from the European Collection of Cell Cultures (ECACC). Human THP-1 cells were cultured in Roswell Park Memorial Institute (RPMI) 1640 medium with 2 mM l-glutamine, 10% foetal bovine serum (FBS) and 50 IU/mL penicillin and 50 μg/mL streptomycin. Cells were maintained at 37 °C with 5% CO_2_ in a humidified incubator. To generate THP-1 differentiated macrophages, cells were stimulated with 320 nM of phorbol 12-myristate 13-acetate (PMA) for 48 h at 37 °C, 5% CO_2_ in a humidified incubator. 

### 4.3. Intracellular Ca^2+^ Measurements

Cells at 2 × 10^5^/well were loaded for 1 h at 37 °C with 2 μM Fura-2 AM. Cells were washed twice with physiological saline (SBS) buffer. Measurements were performed at 37 °C on a 96-well plate reader (FlexStation III, Molecular Devices, Sunnyvale, CA, USA). The change in intracellular Ca^2+^ concentration is indicated as a ratio of fura-2 emission intensities for 340- and 380-nm excitation (F ratio). SBS buffer contained (mM): 130 NaCl, 5 KCl, 1.2 MgCl_2_, 1.5 CaCl_2_, 8 d-glucose, 10 HEPES; pH 7.4. Ca^2+^-free SBS was prepared by excluding CaCl_2_ and supplemented with 2 mM ethylene glycol-bis(β-aminoethyl ether)-*N,N,N߰,N߰*-tetraacetic acid (EGTA). Loading of cells with Fura 2-AM was performed in SBS buffer supplemented with 0.01% (*w/v*) pluronic acid. All compounds were pre-treated onto cells for 30 min before Ca^2+^ measurements. 

### 4.4. RNA Extraction and cDNA Synthesis

Total RNA was isolated from cells using TRI Reagent (Sigma Aldrich, St. Louis, MO, USA) and contaminating genomic DNA was eliminated using DNA-free kit (Ambion, Foster City, CA, USA). cDNA was synthesized from 0.5 μg of total RNA using SuperScript II reverse transcriptase kit (Invitrogen, Waltham, MA, USA).

### 4.5. Quantitative Real-Time PCR

Taqman primer probes sets for human P2X1 (Hs00175686_m1), P2X2 (Hs04176268_g1), P2X3 (Hs01125554_m1), P2X4 (Hs00602442 _m1), P2X5 (Hs01112471_m1), P2X6 (Hs01003997_m1), P2X7 (Hs00175721 _m1), β-actin (Hs01060665_g1) were obtained pre-designed from Applied Biosystems (Foster City, California, USA). qRT-PCR was performed in a 7500 Fast Real-Time PCR instrument (Applied Biosystems). Target gene expression was normalized to β-actin endogenous control and relative quantification was done by the ∆∆*C*t method.

### 4.6. Generating Knockdown P2X4 THP-1 Cells

THP-1 cells (2.5 × 10^5^) were transduced with either scrambled shRNA or P2X4 shRNA lentiviral particles (Thermo Scientific, Loughborough, UK) in the presence of 8 μg/mL hexadimethrine bromide and Lipofectamine 2000 (Thermo Fisher, Loughborough, UK).

### 4.7. Immunocytochemistry

Cells at 2.5 × 10^4^/mL were fixed with 4% paraformaldehyde (15 min, RT) and permeabilized with 0.25% Triton X-100 (10 min, RT). Cells were blocked with 1% bovine serum albumin (30 min, RT) and incubated overnight at 4 °C with primary antibodies (rabbit polyclonal P2X4 (Alomone, Jerusalem, Israel), goat polyclonal P2X1,5,7 (Santa-Cruz biotechnology, Dallas, TX, USA). Cells were stained with secondary antibody goat anti-rabbit (Life Technologies, Waltham, MA, USA) or rabbit anti-goat (Abcam, Cambridge, UK) AF488. Nuclear staining was performed with Vectashield Antifade containing DAPI (Vectorlabs, Peterborough, UK). Cell imaging was performed using laser-scanning confocal microscope Zeiss LSM510 META (Zeiss, Oberkochen, Germany). 

Flow cytometry—Cells (100 μL at 1 × 10^6^/mL) were incubated for 10 min at room temperature (RT) with Fc block (BD, San Jose, CA, USA) and immunostained with anti-human P2X4 (Alomone, Jerusalem, Israel) or hIgG isotype control (Life Invitrogen, Waltham, MA, USA) followed by AlexaFluor 488 (AF488) (Life Invitrogen, Waltham, MA, USA). Cells were acquired on the Cytoflex instrument (Beckman Coulter, Franklin Lakes, NJ, USA). Analyses were performed on CytExpert software (Beckman Coulter, Franklin Lakes, NJ, USA). 

Statistical analysis—Data were analyzed using Origin Pro 9.0 software (Origin Lab, Northampton, MA, USA). Concentration-response curves were fitted assuming a Hill coefficient of 1. Hypothesis testing for experiments with paired datasets were performed by means of paired Student’s *t*-test using Origin Pro 9.0. Data are expressed as mean ± SEM of at least three independent experiments.
